# Metabolomic and functional analyses of small molecules secreted by intestinal nematodes in the activation of epithelial tuft cells

**DOI:** 10.1007/s11306-025-02248-w

**Published:** 2025-04-21

**Authors:** Marta Campillo Poveda, Stephan Löser, Victoria Gillan, Josh Richards, Claire Ciancia, Gavin Blackburn, Erin Kerr, Michael Barrett, Katie A. Hildersley, Philippe Jay, Eileen Devaney, Tom N. McNeilly, Collette Britton, Rick M. Maizels

**Affiliations:** 1 School of Infection and Immunity, University of Glasgow, 120 University Place, Glasgow, G12 8TA UK; 2 School of Biodiversity, One Health and Veterinary Medicine, University of Glasgow, Glasgow, UK; 3 Moredun Research Institute, Penicuik, UK; 4 Institute of Functional Genomics (IGF), University of Montpellier, CNRS, Inserm, Montpellier, France; 5 Present Address: GlaxoSmithKline GmbH, Prinzregentenplatz 9, 81675 Munich, Germany; 6 Present Address: Institute of Evolutionary Biology, University of Edinburgh, Edinburgh, UK

**Keywords:** Excretory-secretory metabolites *Haemonchus contortus*, Malate, *Nippostrongylus brasiliensis*, Undecanoic acid, Untargeted metabolomics

## Abstract

**Introduction:**

Intestinal helminth parasites trigger the host immune response through epithelial sensory tuft cells, but helminth-derived molecules that may activate tuft cells are poorly characterized.

**Objectives:**

The study aimed to identify small molecules released in vitro by two nematode parasites, that infect rodents (*Nippostrongylus brasiliensis)* and ruminants (*Haemonchus contortus*), and to test candidate ligands in an in vivo model of tuft cell differentiation.

**Methods:**

Small molecules were analyzed by hydrophilic interaction liquid chromatography (HILIC) of material released by adult parasites incubated in serum-free media, followed by mass spectrometry; selected molecules were administered to mice and tuft cell expansion enumerated after 5 days.

**Results:**

A range of different conditions (culture media, timing, oxygenation) were tested, and comparisons made between the conditions, and between the two nematode species at selected points. Common products across the conditions and species included carboxylic acids (malate, succinate), medium chain fatty acids (such as decanoic and undecanoic acids), purines (guanine, xanthine and their derivatives), and phosphocholine compounds. We selected 19 of the prominent molecules for in vivo testing by oral administration, including succinate, a known activator of tuft cell differentiation. Malate elicited a low but significant level of tuft cell expansion, while undecanoic acids with or without a bromine substitution were also able to induce significant differentiation comparable to succinate. Other molecules including phosphorylcholine had no effect.

**Conclusion:**

Multiple molecular species including decanoic and undecanoic acids released by helminths may contribute to activation of tuft cells in vivo.

**Supplementary Information:**

The online version contains supplementary material available at 10.1007/s11306-025-02248-w.

## Introduction

Helminth parasites and the mammalian immune system have co-adapted through long evolutionary time, with elaborate parasite immune evasion strategies matched by a fine-tuned host immune system to detect and eliminate parasites (Douglas et al., 2021; Girgis et al., 2013; Maizels & Gause, 2023). Nonetheless, helminth infections present a major health burden across the tropics with an estimated 1.45 billion infections with soil-transmitted helminths alone (Pullan et al., 2014), while livestock infestations inflict enormous economic losses across the world (Charlier et al., 2020; Roeber et al., 2013).

The mammalian immune system has established multiple mechanisms in response to helminth parasites, broadly classed as type 2 immunity (Hammad et al., 2022; Lloyd & Snelgrove, 2018; Maizels & Gause, 2023). Responder cells at barrier surfaces release alarmins and cytokines that signal the infection to other immune cells, including type-2 innate lymphoid cells (ILC2s), alternatively activated macrophages (M2s) and eosinophils (Grencis, 2015; McDaniel et al., 2023; Zaiss et al., 2024). Once adaptive immunity has been induced, antigen-specific Th2 and B cells mobilise to reduce parasite burdens or expel worms completely (Harris & Loke, 2017; Maizels & Gause, 2023).

Notably, expulsion of gastrointestinal (GI) parasites is dependent on a fully-responsive epithelial population. Thus, goblet cells, instructed by IL-13-producing ILC2s and Th2 cells, execute anti-helminth effects by secreting mucus and resistin-like molecule β (RELM-β) (Finkelman et al., 2004; Herbert et al., 2009). More recently, another epithelial cell type, the tuft cell, has been recognized as instrumental in activating the immune response against helminths and protists, with the intestinal nematode *Nippostrongylus brasiliensis* being a particularly potent stimulus for tuft cell expansion (Gerbe et al., 2016; Howitt et al., 2016; Kotas et al., 2023; von Moltke et al., 2016). Tuft cells are also endowed with critical effector functions (Billipp et al., 2020; Campillo Poveda et al., 2023), including the production of acetylcholine which acts in the intestinal tract to increase fluid egress (Billipp et al., 2024) and directly compromise helminth neurological function (Ndjim et al., 2024).

Tuft cells, characterised by an apical tuft-like structure of microvilli reaching into the intestinal lumen, are chemosensory cells expressing a range of G-protein coupled receptors (GPCRs) including small molecule taste sensors, signalling through the cation channel Trpm5 (Billipp et al., 2020; Campillo Poveda et al., 2023; O’Leary et al., 2019). In the intestinal tract, tuft cells are crucial components of the anti-helminth immune response as tuft cell-deficient *Pou2f3*^–/–^ mice are unable to clear an acute *Nippostrongylus brasiliensis* roundworm infection (Gerbe et al., 2016). In response to infection, tuft cells release IL-25 that activates IL-13 production by ILC2s, creating a positive feed-forward loop that drives goblet and tuft cell hyperplasia for parasite expulsion (Gerbe et al., 2016; von Moltke et al., 2016). Similar expansion of tuft cells is found in ruminants infected with GI nematodes (Hildersley et al., 2021), demonstrating the evolutionary conservation of this pathway.

Infection with the protist *Tritrichomonas muris* also induces a tuft cell response, through Trpm5 activation (Howitt et al., 2016) involving the succinate GPCR (Sucnr1/GPR91) (Nadjsombati et al., 2018; Schneider et al., 2018). Succinate administration via drinking water directly drives Sucnr1-dependent tuft cell and subsequent ILC2 activation in distal parts of the small intestine (Lei et al., 2018; Nadjsombati et al., 2018). Although both the protist *T. muris* and the helminth *N. brasiliensis* have been shown to produce succinate (Nadjsombati et al., 2018), *Sucnr1*-deficient mice were incapable of clearing the protist infection, but were able to expel *N. brasiliensis* worms even in the absence of Sucnr1 (Lei et al., 2018; Nadjsombati et al., 2018), suggesting redundancy and involvement of additional receptors.

Tuft cells express bitter taste receptors of the Tas2r family, some of which are upregulated in helminth infection, and stimulation with bitter compounds induces IL-25 release (Luo et al., 2019), although it is not known whether Tas2r receptors are required for parasite immunity. Tuft cells also express the sweet/umami taste receptor Tas1r3, although this receptor is not required for tuft cell responses to another intestinal nematode, *Heligmosomoides polygyrus* (Howitt et al., 2020). The receptor(s) activating tuft cells in helminth infection therefore remain unclear and any helminth-derived metabolites driving tuft cell activation have yet to be identified.

Some indications of possible ligands arise from analyses of other micro-organisms, and from studies on nematode biological interactions. In the case of *Shigella* bacterial infection, the metabolite *N*-undecanoylglycine was found to activate tuft cells, and to do so via the vomeronasal GPCR Vmn2r26 that is associated with olfactory sensing (Xiong et al., 2022). In the helminth setting, a widely represented small molecule family is the ascarosides, small dideoxy sugars with short chain fatty acid moieties, which function as pheromone signals between individual nematodes (Choe et al., 2012). Among these, ascarosides released from *N. brasiliensis* adult worms mediate immunosuppressive effects in mouse models of inflammation (Manosalva et al., 2015; Shinoda et al., 2021).

For tuft cells to function as sentinels of infection, they must sense products released by incoming parasites; proteomics has identified excretory/secretory proteins from helminths, released during in vitro culture (Harnett, 2014; Hewitson et al., 2009), but only recently has attention turned to the small molecule repertoire exported from helminth parasites (Chen et al., 2021; Wangchuk et al., 2019a, 2019b, 2019c; Whitman et al., 2021; Yeshi et al., 2020), recently reviewed by Wangchuk (Wangchuk et al., 2023). Analyses of small molecules released both by adult worms (Wangchuk et al., 2019b) and infective L3 larvae of *N. brasiliensis* (Yeshi et al., 2020), as well as the intestinal contents of infected mice (Chen et al., 2021), identified 45–55 polar metabolites and several hundred lipid species.

In searching for possible tuft cell ligands, we surmised that the host system evolved to sense the presence of molecules common to multiple helminth species, possibly resulting from an obligatory metabolic pathway that is physiologically essential to the parasites. Such a product may be, like succinate, a familiar molecule which activates at high concentrations, or a novel component foreign to the mammalian system that can be more sensitively detected. To pursue this inquiry, we conducted parallel metabolomic analyses on two major GI nematode parasites, the laboratory model mentioned above, *N. brasiliensis,* and the ruminant “barber’s pole” roundworm *Haemonchus contortus.* While the metabolic biochemistry of these species has been well described (Harder, 2016), this has been at the whole-organism level rather than, as we report here, a focus on released molecules which may interact with the host.

## Methods

### Animals and parasites

*N. brasiliensis* was maintained in rats as previously described (Camberis et al., 2003). *H. contortus* (MHco3(ISE) strain) was recovered from infected sheep as previously described (Laing et al., 2013). Sheep were aged four to nine months and were reared and housed indoors under parasite-free conditions prior to *H. contortus* infection. Parasite maintenance and animal experiments in which mice received metabolites in drinking water were conducted under a UK Home Office licence and approved by the University of Glasgow Animal Welfare and Ethical Review Board (AWERB). All experimental procedures in sheep were examined and approved by the Moredun Research Institute AWERB and were conducted under approved UK Home Office licence in accordance with the 1986 Animal (Scientific Procedures) Act, UK.

### Parasite cultures

*N. brasiliensis* adult worms were incubated at a concentration of 90–200 worms/mL in Earle’s Balanced Salt Solution (EBSS, GIBCO 24010-043), Hanks’ BSS (HBSS, Sigma H8264), or RPMI1640 (Sigma R7509), each with gentamycin (1/1000 dilution; Sigma G1397, 50 mg/mL); each culture condition was set up as 1 mL volumes in 3 separate wells of a 48-well plate, with additional control wells containing media without worms. Worms were then incubated at 37C° for 4 or 24 h. Under normoxic conditions, cultures were in 5% CO_2_ in air; under hypoxic conditions a mixture of 1% O_2_ 5% CO_2_ 94% N_2_ was used for 4 or 24 h inside an airtight Billups-Rothenberg hypoxic chamber.

*H. contortus* adult worms were washed 6 times in warm HBSS followed by 3 washes in HBSS + penicillin/streptomycin (1/1000 dilution; Invitrogen 15140-122, 10,000 units/mL). Worms were then washed in the respective media (HBSS, EBSS or RPMI1640) and male and female worms cultured separately at a concentration of 10 worms/mL as described for *N. brasiliensis*. For both parasites, media were collected at 4 and 24 h and immediately quenched in a dry ice/ethanol bath before centrifuging in a chilled microfuge at 1900 g for 10 min.

### Metabolomic analyses

Analyses were performed on 10 µL of culture supernatant, to which was added 200 μL chloroform:methanol:water (CMW, 1:3:1) extraction solvent at 4 °C. Following mixing, samples were centrifuged at 13,000 g, 4 °C for 5 min, and the supernatants (~ 195 μL) transferred to fresh 2 mL screw-top Eppendorf tubes, prior to displacing air with argon gas. Samples were stored at -80 °C until analysis.

Samples were subjected to hydrophilic interaction liquid chromatography (HILIC) on a Dionex UltiMate 3000 RSLC system (Thermo Fisher Scientific) using a ZIC-pHILIC column (150 mm x 4.6 mm, 5 μm column, Merck Sequant) maintained at 40 °C and eluted with a linear gradient of 20 mM ammonium carbonate (20% to 95%) versus acetonitrile (80% to 5%) at a flow rate of 0.3 mL/min for 20 min.

Fractions were then injected into an Orbitrap Fusion (Thermo Fisher Scientific), operated in polarity switching mode with the following settings: Resolution 120,000; AGC 2e5; m/z range 70–1000; Sheath gas 40; Auxiliary gas 5; Sweep gas 1; Probe temperature 150 °C; Capillary temperature 325 °C. For positive mode ionization: source voltage + 4.3 kV. For negative mode ionization: source voltage − 3.2 kV. S-Lens RF Level 60.00%.

Annotated data files were analyzed using the online service of Metabolanalyst 5.0 (https://www.metaboanalyst.ca/), R studio and Prism for comprehensive data normalization, statistical and functional analyses.

### In vivo administration of metabolites

Metabolites administered were: L-alanine (A7627, Sigma-Aldrich); 7-aminomethyl-7-carbaguanine (PreQ1 dihydrochloride, SML0807, Sigma-Aldrich); L-carnitine hydrochloride (C0283, Sigma-Aldrich), creatine (C0780, Sigma-Aldrich); *N*^G^-*N*^G^-dimethyl-L-arginine (D0390, Sigma-Aldrich); 1,2-dioleoyl-sn-glycero-3-phosphocholine (P6354, Sigma-Aldrich); hypoxanthine (H9377, Sigma-Aldrich), 7- and 9-methylguanine (67073, 67074, Sigma-Aldrich); malic acid (M1000, Sigma-Aldrich); phosphocholine chloride (P0378, Sigma-Aldrich); pyruvic acid (107360, Sigma-Aldrich); uridine (U6381, Sigma-Aldrich), xanthine (X7375, Sigma-Aldrich), 3-methylguanine (sc-483803, Santa Cruz Biotechnology), undecanoic acid (171476, Sigma-Aldrich), 10-bromodecanoic acid (541397, Sigma-Aldrich) and 11-bromoundecanoic acid (B82804, Sigma-Aldrich). Details of concentration, delivery route and timing are detailed in the relevant Figure Legends.

### Immunohistochemistry

C57BL/6 female mouse small intestines were embedded in paraffin using the Swiss-rolling technique (Bialkowska et al., 2016), which enables multiple villi to be investigated in a single cut section. Transverse sections were made using a microtome through the gut rolls at a thickness of 5 µm before mounting on glass slides. Sections were deparaffinized by immersing slides in xylene, then hydrated through 100%, 90%, and 70% ethanol successively. Heat-induced epitope retrieval was performed in 10 mM citrate buffer pH 6 (Thermo Fisher Scientific), and then sections were blocked using 2.5% normal horse serum blocking solution (Vector Laboratories) for 1 h at room temperature. Slides were then incubated overnight at 4 °C with rabbit anti-mouse Dclk1 antibody (Abcam) diluted 1/1000 in 2.5% normal horse serum blocking solution. Polyclonal rabbit IgG (Abcam) was used as an isotype control. After washing, sections were incubated with swine anti-rabbit-IgG/FITC (Agilent Dako), washed, and mounted using Vectashield Vibrance antifade mounting medium with DAPI (Vector Laboratories). Slides were imaged using a Leica DMi8 inverted microscope and Leica Application Suite (LAS) X software (Leica Microsystems). The resulting image files were analyzed using ImageJ/Fiji. For each sample, 5 areas that included 5 villus-crypt units were randomly selected, and the number of stained tuft cells counted; from this the number of tuft cells per villus-crypt axis for each mouse was recorded.

### Statistics

Functional annotation of distinguishing metabolites was performed using GraphPad Software (Boston, Massachusetts USA) and RStudio Version 2023. Statistical analysis generated q-values, which provide a more accurate estimate than p values of the false discovery rate (FDR) when dealing with multiple comparisons. Tuft cell numbers from the in vivo experiments were analyzed using a one-way ANOVA test with multiple comparisons with normality and lognormality tests of tuft cell counts.

## Results

**Fig. 1:**
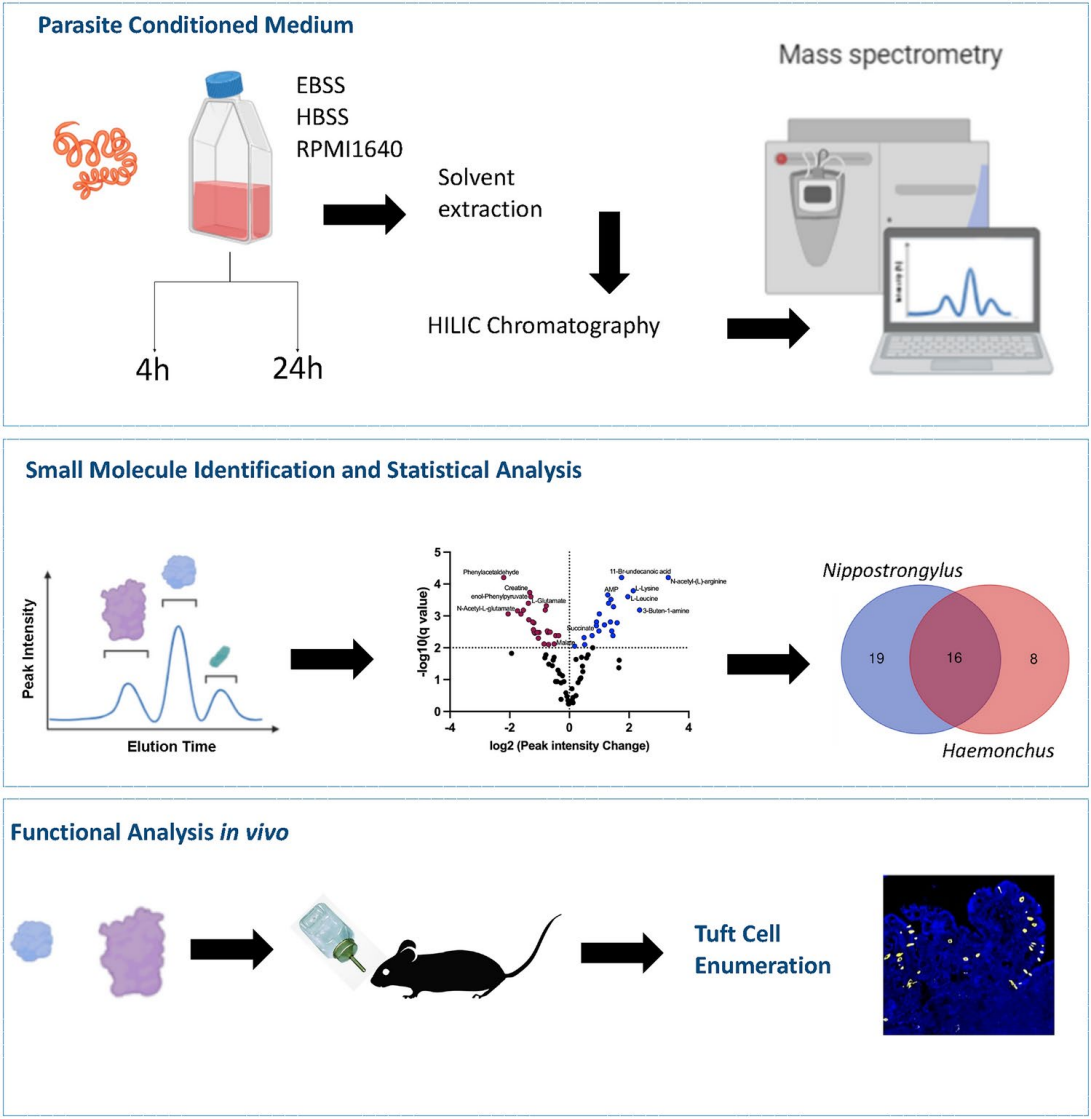
Methodology of small molecule analysis. Intestinal nematodes (*H. contortus* and *N. brasiliensis* adult worms) were cultured under various conditions for 4–24 h. Conditioned media were collected; small molecules extracted into chloroform/methanol and subject to mass spectrometry; identified peaks were compared and statistically verified; individual molecules of interest were administered to mice in drinking water to test for induction of tuft cells, enumerated by histological staining for the DCLK1 marker

Analyses of small molecules was undertaken by hydrophilic interaction liquid chromatography (HILIC) of material released by adult parasites incubated in serum-free media, followed by mass spectrometry.. A range of different conditions (culture media, timing, oxygenation) were tested, and comparisons were made between the conditions, and between the two nematode species, as described below. In addition, the major metabolites were administered in vivo to experimentally determine effects on tuft cell induction. An overview of the study is shown in Fig. 1.

### Culture media and metabolite profiles for adult *N. brasiliensis*

We first explored optimal culture conditions for small molecule identification. In collecting excretory-secretory proteins from helminths, most protocols have used nutrient-enriched media that allows survival for days or even weeks in vitro (Harnett, 2014; Hewitson et al., 2009). For the purposes of collecting small molecules released over shorter time-windows, simpler conditions were selected. We compared incubation of adult worms at 37 °C in EBSS, HBSS or RPMI1640 tissue culture media. While BSS media are simply isotonic buffered salt solutions, RPMI1640 contains all 20 amino acids and 11 vitamins required for longer-term cell survival in tissue culture.

**Fig. 2:**
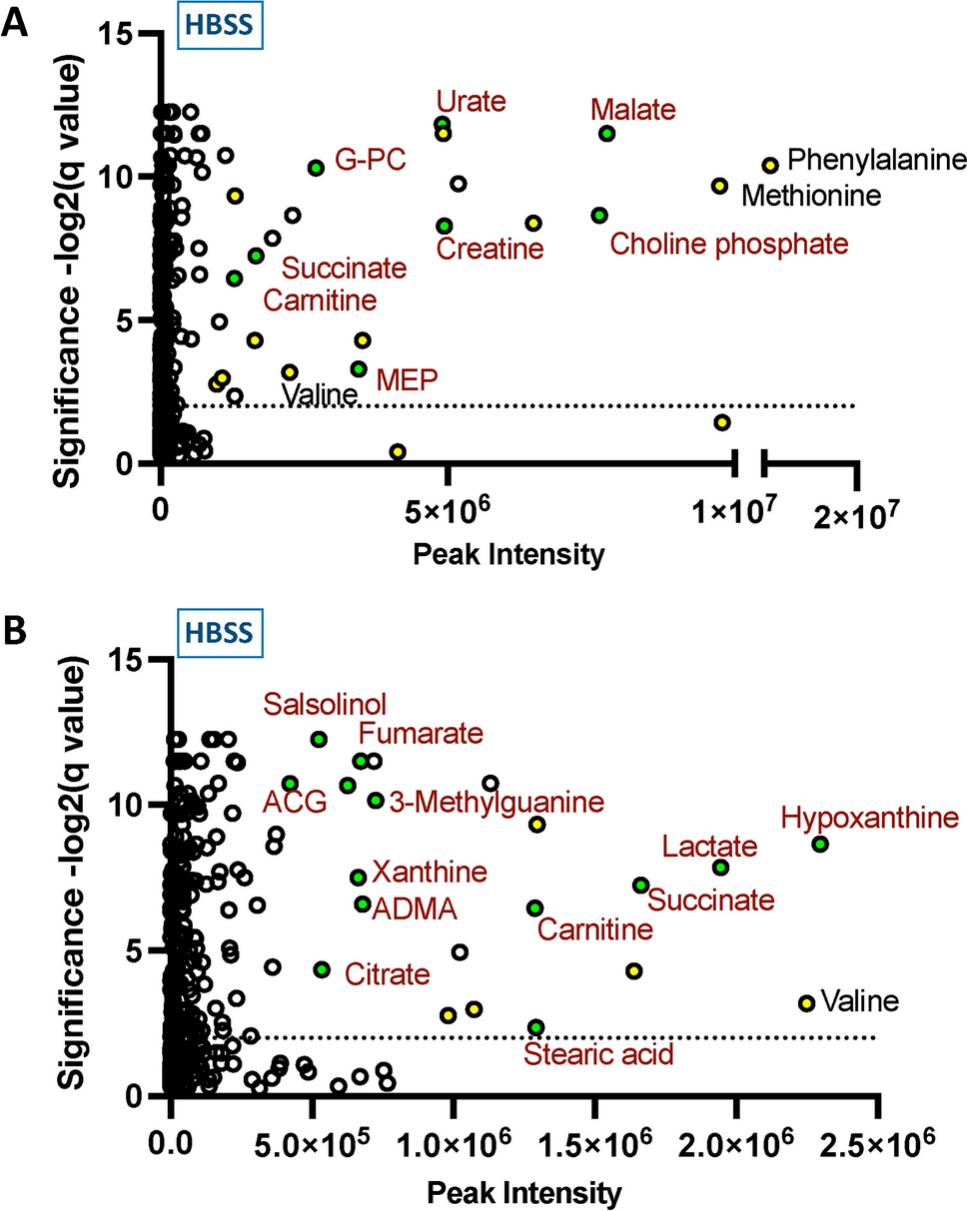
Small molecules released by *N. brasiliensis* adult worms. In each case, 200 adults were incubated in 1 mL media; after 4 h, supernatants were recovered and extracted in chloroform:methanol:water for MS analysis. **A** Graphical plot of metabolites released in HBSS at 4 h showing peak intensities (with medium alone subtracted). Amino acids Met, Phe and Val are annotated, and together with all others (Arg, Glu, His, Leu, Lys, Pro, Trp, Tyr) coloured yellow. Named molecules coloured green. G-PC, sn-Gycero-3-Phosphocholine; MEP, 2-C-Methyl-D-Erythritol 4-Phosphate. Stearic acid is octadecanoic acid. **C** As A but expanded x axis from 0–2.5 × 10^6^, and coloured as in **A** ACG, 7-aminomethyl-7-carbaguanine,. ADMA asymmetric dimethylarginine. Stearic acid is octadecanoic acid

For each media type, three replicate parasite cultures were incubated and supernatants recovered after 4 h. Parasites cultured in either EBSS or HBSS released a greater number of metabolites identified above the significance threshold than parasites cultured in RPMI1640. This was in part due to the richer set of constituents in RPMI1640, which resulted in a high baseline for those metabolites. For example, the inclusion of choline chloride in RPMI1640 evidently confounded identification of this and related molecules. The greatest number of identifications were made in HBSS (Fig. 2 A, B), most of which were also found in EBSS, and many in RPMI1640-derived samples (Table 1).

**Table 1:** Peak intensities are calculated after subtraction of values from medium alone controls; nd denotes values not significantly different from medium alone

		HBSS Peak intensity	HBSS -log2 (q value)	EBSS Peak intensity	EBSS -log2 (q value)	RPMI1640 Peak intensity	RPMI1640 -log2 (q value)
1	L-Phenylalanine	1.72E + 07	10.63	2.83E + 06	10.39	7.91E + 04	11.64
2	L-Proline	9.79E + 06	12.67	nd	nd	2.49E + 04	1.669
3	L-Methionine	9.74E + 06	12.27	9.79E + 05	9.68	nd	nd
4	**(S)-Malate**	7.77E + 06	5.797	1.30E + 06	11.50	5.58E + 05	12.9
5	**Choline phosphate**	7.64E + 06	6.712	1.39E + 06	8.65	2.89E + 04	4.624
6	L-Histidine	6.50E + 06	12.99	1.67E + 06	8.39	1.02E + 04	3.076
7	N-Formyl-L-methionine	5.19E + 06	4.523	5.96E + 05	9.76	2.99E + 07	12.13
8	**Creatine**	4.94E + 06	HB	8.28E + 04	4.34	4.72E + 04	1.071
9	L-Glutamine	4.93E + 06	12.71	6.69E + 05	11.50	2.60E + 03	10.49
10	Urate	4.91E + 06	6.981	1.34E + 06	11.83	1.65E + 04	1.722
11	L-Arginine	4.13E + 06	12.71	nd	nd	nd	nd
12	L-Leucine	3.52E + 06	12.75	nd	nd	2.52E + 03	11.08
13	2-C-Methyl-D-erythritol 4-phosphate (MEP)	3.45E + 06	8.17	nd	nd	2.12E + 02	8.27
14	**sn-Glycero-3-phosphocholine**	2.70E + 06	5.572	5.64E + 05	10.30	nd	nd
15	**Hypoxanthine**	2.30E + 06	5.308	2.46E + 05	8.65	nd	nd
16	L-Valine	2.25E + 06	12.99	nd	nd	2.09E + 03	0.9385
17	(R)-Lactate	1.95E + 06	3.182	6.34E + 05	7.86	7.48E + 04	9.617
18	**Succinate**	1.66E + 06	3.905	2.07E + 05	7.25	2.14E + 04	13.78
19	L-Tyrosine	1.64E + 06	12.49	nd	nd	1.47E + 03	4.686
20	L-Tryptophan	1.30E + 06	12.75	2.51E + 05	9.33	3.02E + 04	6.686
21	Stearic (octadecanoic) acid	1.29E + 06	4.124	nd	nd	3.62E + 04	4.513
22	L-**Carnitine**	1.29E + 06	5.569	1.23E + 05	6.46	2.45E + 03	7.627
23	Choline	1.13E + 06	11.61	1.13E + 05	10.74	nd	nd
24	L-Glutamate	1.07E + 06	10.62	nd	nd	nd	nd
25	Pentanoate	1.02E + 06	1.977	2.26E + 05	4.95	nd	nd
26	L-Lysine	9.82E + 05	10.75	nd	nd	nd	nd
27	Picolinamide	7.66E + 05	12.68	nd	nd	8.38E + 02	3.263
28	Sodium dodecyl sulfate	7.53E + 05	1.802	nd	nd	1.61E + 04	2.678
29	**3-Methylguanine***(Note 1)*	7.25E + 05	7.758	1.39E + 05	10.15	6.06E + 04	11.13
30	3-(4-Hydroxyphenyl)lactate	7.18E + 05	5.033	6.28E + 04	11.50	5.53E + 06	10.22
31	**NG,NG-Dimethyl-L-arginine**	6.79E + 05	7.126	nd	nd	4.05E + 03	8.156
32	Fumarate	6.72E + 05	5.649	1.10E + 05	11.50	5.48E + 06	11.71
33	5-Hydroxyindoleacetaldehyde	6.69E + 05	2.746	2.38E + 06	0.67	8.87E + 03	10.44
34	**Xanthine**	6.63E + 05	5.572	3.03E + 04	7.51	1.40E + 04	2.344
35	1-Methylhypoxanthine	6.25E + 05	8.335	1.65E + 05	10.67	1.74E + 05	10.22
36	Triethyl phosphate	5.93E + 05	0.5901	nd	nd	9.95E + 03	5.61
37	Citrate	5.35E + 05	1.409	8.28E + 04	4.34	2.79E + 03	1.25
38	Salsolinol	5.24E + 05	5.312	1.47E + 04	12.26	nd	nd
39	Hexanoic acid	4.86E + 05	3.124	1.80E + 06	0.83	7.16E + 03	1.571
40	Hordatine A	4.72E + 05	1.573	7.58E + 04	1.13	nd	nd
41	**7-Aminomethyl-7-carbaguanine**	4.22E + 05	7.09	1.11E + 05	10.73	2.10E + 05	11.78
42	**L-Alanine**	3.83E + 05	6.546	nd	nd	3.12E + 05	11.58
43	Phosphodimethylethanolamine	3.73E + 05	5.788	8.41E + 04	9.00	1.59E + 05	11.87
44	Nicotinate	3.66E + 05	4.225	7.30E + 04	8.58	nd	nd
45	Piperidine	3.60E + 05	12.8	nd	nd	1.72E + 04	3.921
46	Hexadecanoic acid	3.58E + 05	4.942	nd	nd	4.40E + 02	4.07
47	L-Threonine	3.14E + 05	12.45	nd	nd	nd	nd
48	3,4-Methylenedioxymethamphetamine	3.06E + 05	1.848	nd	nd	3.22E + 03	0.8547
49	Phenylacetaldehyde	2.88E + 05	2.345	nd	nd	nd	nd
50	2’,4’,6’-Trihydroxydihydrochalcone	2.83E + 05	0.5813	nd	nd	2.44E + 02	10.23

RPMI1640 contains 3 µg/mL choline chloride, as well as vitamins and all 20 amino acids resulting in high background levels for those constituents. ***Bold*** indicates compounds tested in vivo (Fig. 5)

Note 1: may represent 3-, 5- or 7-methylguanine with identical mass values

A number of amino acids were amongst the most abundant molecules, most notably phenylalanine and methionine (indicated with yellow symbols in Fig. 2 A, B). Other than amino acids, a number of purine precursors and derivatives, and amino acid metabolites, were noted as previously reported for *N. brasiliensis* (Chen et al., 2021). Among the purine derivatives was methylguanine, that may be related to the 5′ cap of mRNA that is often trans-spliced with a trimethyl (2′2′7′-trimethylated) guanine in nematodes (Thomas et al., 1988). A few citric acid and tricarboxylic acid cycle metabolites were found, most conspicuously malate, and succinate. Both a short-chain (valeric acid) and long-chain (stearic acid) fatty acids were identified, the former having been reported as interfering with histone deacetylation required for CD4^+^ T cell activation (Luu et al., 2019). We also identified high levels of choline phosphate (phosphocholine) which is widely expressed across a spectrum of nematode parasites (Harnett et al., 1993; Maizels et al., 1987; Péry et al., 1979).

Identifying metabolites in parasite cultures depended on comparison with a standardised set of controls for mammalian and bacterial metabolism, therefore we were not able to conclusively identify ascarosides that are known to be released by nematodes (Choe et al., 2012; Shinoda et al., 2021). However, a prominent peak with mass 276 Daltons was noted in both EBSS- and HBSS-cultured media that is likely to represent ascr#1 (Supplementary Fig. 1).

### Metabolite profiles of* Haemonchus contortus* culture media

**Fig. 3:**
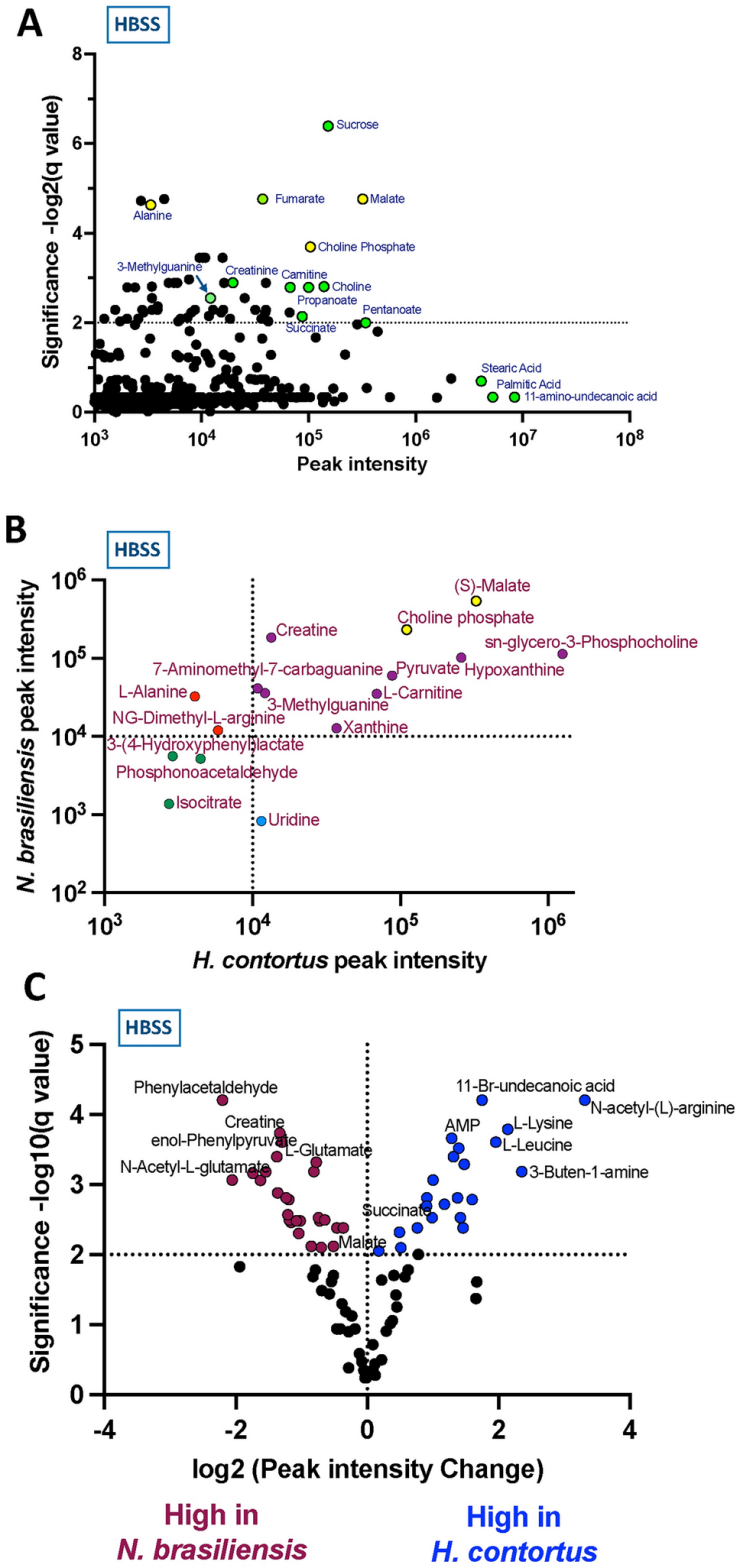
Metabolites from *H. contortus* adult worms and comparison with those from *N. brasiliensis*. Plots of molecules identified from *H. contortus* incubated in HBSS. Amino acids are coloured yellow (only Ala is annotated), selected identified metabolites coloured green. NB, palmitic acid is hexadecanoic acid, stearic acid is octadecanoic acid. **A.** Plots of molecules identified from *H. contortus* incubated in HBSS. Amino acids are coloured yellow (only Ala is annotated), selected identified metabolites coloured green. NB, palmitic acid is hexadecanoic acid, stearic acid is octadecanoic acid. **B** Comparative intensity of 15 major metabolites released by the two species. NB Caproic acid is hexanoic acid. **C** Volcano plot comparison of molecules released by the two species with FDR at 0.5

We next analysed another intestinal nematode species, *H. contortus* that colonises the abomasum (true stomach) of ruminant livestock, and is, like *N. brasiliensis*, a member of the Trichostrongyloid superfamily. Adult worms were incubated in EBSS, HBSS or RPMI1640 under similar conditions, for 4 h. As observed for *N. brasiliensis*, fewer molecules surpassed the significance threshold in RPMI1640. A plot of the individual metabolites identified in HBSS is presented in Fig. 3** A**.

Many of the identified molecules from *H. contortus*, including choline compounds, malate and succinate, mirrored those found to be released from *N. brasiliensis* as described in more detail below. However, longer chain fatty acids (palmitic and stearic) were more prominent albeit at a low level of statistical significance in supernatants of *H. contortus.* We also noted the presence of bromo-undecanoic acid in cultures of this parasite.

### Comparative analysis of two helminth species

Metabolomic data generated from analyses of conditioned media from cultures with *N. brasiliensis* and *H. contortus* were combined to identify products released by both species. These analyses identified many common metabolites released into HBSS medium (Fig. 3 B) with similar profiles of small molecules in EBSS and RPMI1640 cultures (Suppl. Figure 3 A, B) Quantitatively, most common products were of similar intensity in the two species, however, some exceptions were noted. While malate and choline compounds (indicated in yellow) were present at high levels for both species, some molecules such as creatine were more prominent in *N. brasiliensis* conditioned media. A volcano plot comparing metabolomic data from the two species, showing the ratios for each molecule, reveals some key findings such as the presence of succinate and the relative abundance of medium chain undecanoic acids in the supernatants from *H. contortus* parasites (Fig. 3 C).

### Time-dependent release of metabolites

**Fig. 4:**
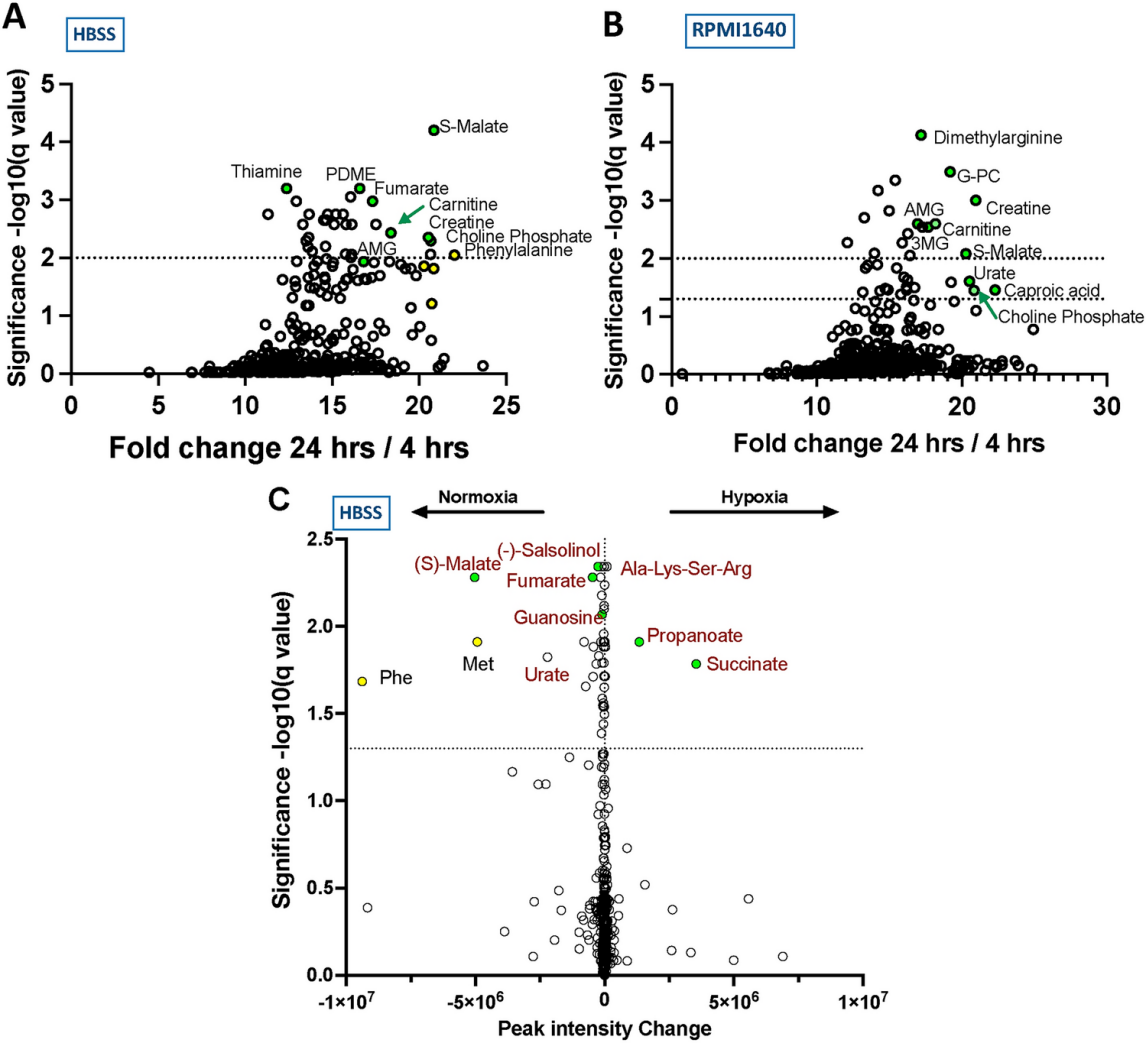
Analysis of small molecules released by *N. brasiliensis* adult worms between 4 and 24 h, and under different oxygenation conditions. In each case, 90 adults were incubated in 1 mL media; after 4 h, supernatants were replaced with fresh media, and conditioned media collected at 24 h; both 4 h and 24 h media were analysed and compared. **A** Fold-change increases in peak intensity from 4 to 24 h in HBSS. **B** As A, in RPMI1640. **C** Comparison of products released under normoxia or hypoxia (1% O_2_) in HBSS

We then compared metabolomic data from *N. brasiliensis* conditioned media collected after 4 h and 24 h incubation; products that showed a significant increase over this time period are less likely to have been carried over by the parasites from their intestinal environment. In this setting we employed both HBSS and RPMI160, as the latter medium is more conducive to maintaining parasite viability. As shown in Fig. 4 A (for HBSS) and B (for RPMI1640), most of the key molecules identified at 4 h were significantly increased after 24 h; these included malate, choline phosphate and guanine derivatives; in the case of RPMI1640, *N. brasiliensis* parasites also elaborated asymmetric dimethylarginine (ADMA), a reported inhibitor of nitric oxide synthase.

### Effects of hypoxia on the release of metabolites

As intestinal parasites are typically found in low-oxygen environments, we then asked whether the profile of small molecule release was affected by manipulation of oxygen levels, with parallel cultures set up under normoxia or hypoxia (1% O_2_). The principal effect of hypoxia was reduced production of numerous metabolites, with the exception of succinate and propanoate, indicating that while *N. brasiliensis* responds to differential oxygenation conditions, it shows a more active general release of metabolites in normoxia than hypoxia (Fig. 4 C).

### In vivo effects on tuft cell induction

**Fig. 5:**
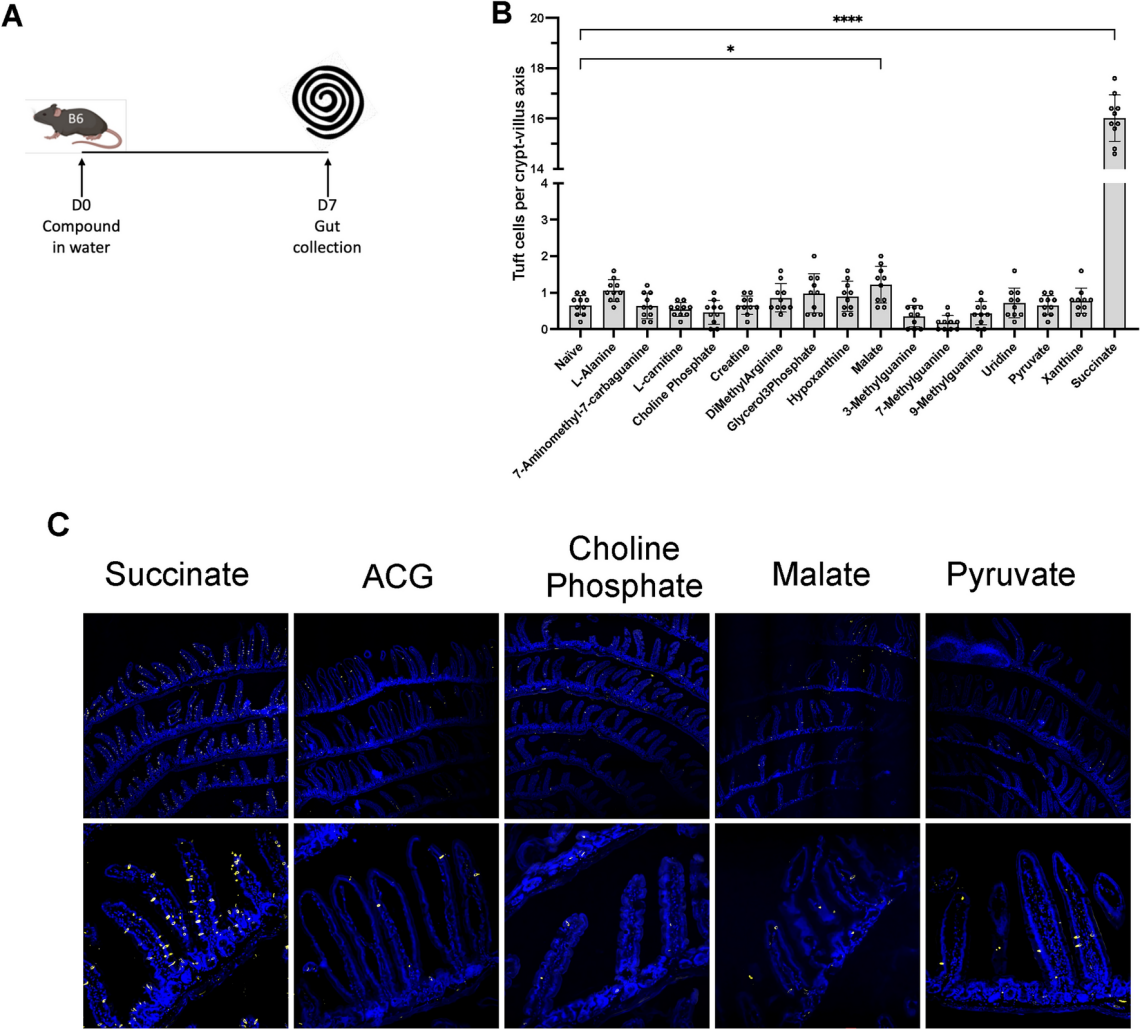
Effect of small molecules on tuft cell differentiation in vivo. **A** Protocol for administration in drinking water. **B** Tuft cell counts, data pooled from two biological replicate experiments (n = 5 mice per experiment, each mouse count based on 5 randomly selected sections). Tuft cell numbers were analysed using one-way ANOVA test with multiple comparisons and Normality and Lognormality Tests for data distribution. **C** Staining for tuft cells in sections from mice treated with succinate (100 mM), 7-amino-methyl-7-carbaguanine (ACG, 10 mM), choline phosphate (20 mM), malate (100 mM) and pyruvate (100 mM). Sections stained at day 7 for DCLK1 expression. Yellow = DCLK1 and blue = DAPI. Scale bars represent 100 µm in all panels. *, p < 0.05, **** p < 0.0001

The major small molecule metabolites that had been identified in this study were then tested by administration in drinking water (Fig. 5 A), using protocols that had previously been shown to induce intestinal tuft cell differentiation in mice (Drurey et al., 2022; Nadjsombati et al., 2018).

The concentrations chosen for each of the metabolites were based on their known safe levels regarding toxicity in rats, as reported on www.drugbank.com, and their solubility in water at 20 °C. Of 14 metabolites listed in Table 1 (3 of which were isomers of methylguanine) and tested in vivo, only succinate was able to drive marked expansion of tuft cell numbers (*p* < 0.0001), although malate did induce a small but significant increase in DCLK-1^+^ tuft cell numbers (*p* < 0.05, Fig. 5B), together with generalized disruption of the epithelial layer (Fig. 5 C). In addition, we tested uridine and pyruvate as less prominent but ubiquitous metabolites, and similarly found no induction of tuft cell expansion.

**Fig. 6:**
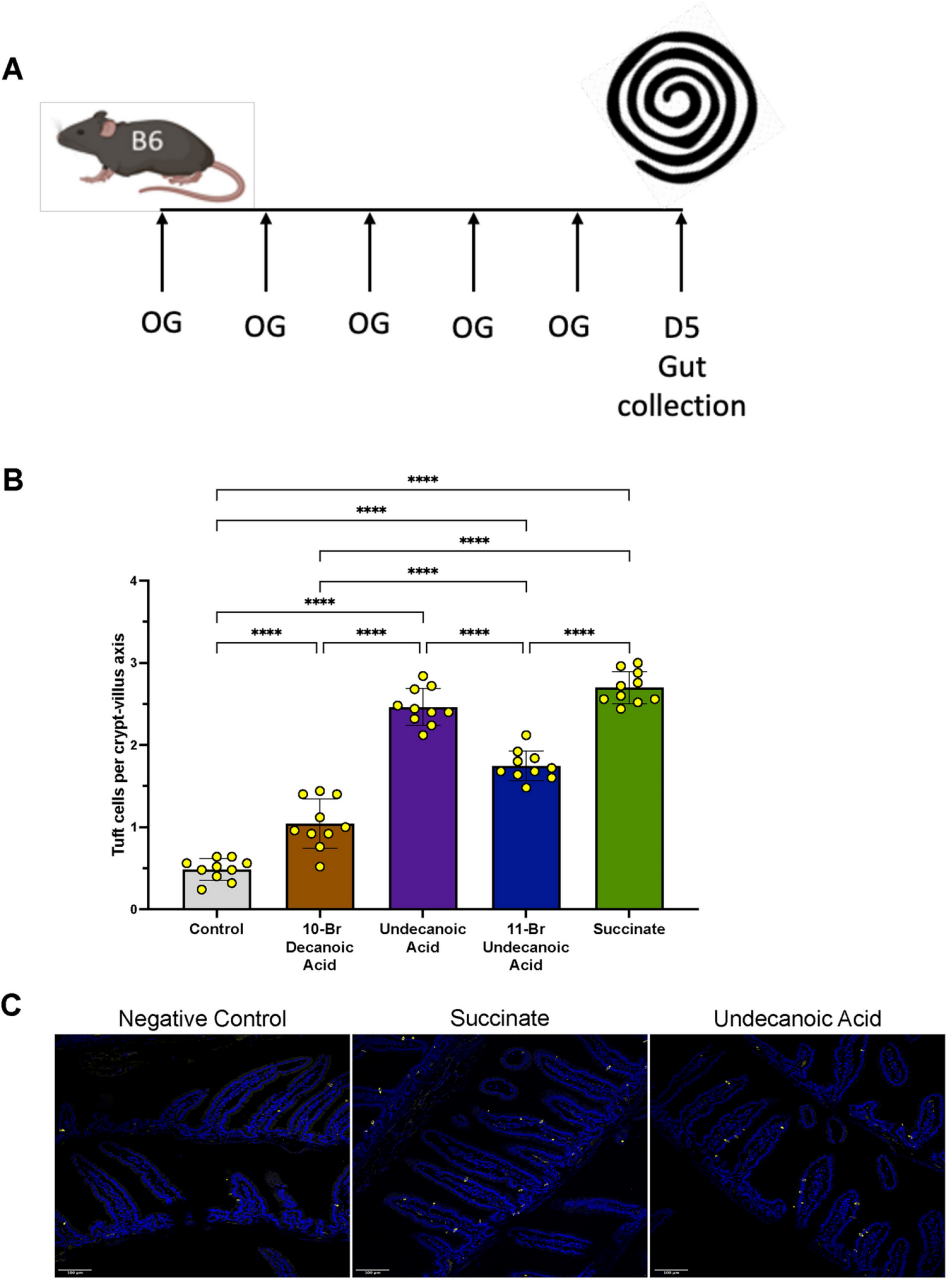
Effect of medium chain fatty acids on tuft cell differentiation in vivo. **A** Protocol for administration by oral gavage (OG). **B** Staining for tuft cells in sections from mice treated with indicated molecules, data pooled from two biological replicate experiments (n = 5 mice per experiment). Tuft cell numbers were analysed using one-way ANOVA test with multiple comparisons and Normality and Lognormality Tests for data distribution. **C** Sections stained for DCLK1 in from mice treated for 5 days with succinate, undecanoic acid and negative control. **** p < 0.0001

Furthermore, the medium-chain (10- and 11-carbon) fatty acids decanoic and undecanoic acid were also present in the identified molecules, in some cases brominated, although the type of substitution cannot be determined from mass values. As these compounds are not soluble in water, decanoic acid and two brominated variants conjugated to C-10 or C11, were administered by oral gavage as previously described by Shoji et al. (Shoji et al., 2022), applying compounds daily for 5 days (Fig. 6 A). This shorter treatment, and its mode of delivery, is clearly not as effective as drinking water administration, as shown by the lower numbers of tuft cells induced with succinate (Fig. 6 B). However, both 10- and 11-carbon compounds stimulated tuft cell expansion when given to mice under these conditions, with undecanoic acid activating the same response as succinate (Fig. 6 B, C), implicating medium-chain fatty acids in tuft cell hyperplasia.

## Discussion

The molecular “arms race” between host and parasite has primarily been conceived at the level of macromolecules, in particular protein components (Maizels et al., 2018). However, small molecules from parasites are increasing in prominence through systematic analyses on metabolite production (Wangchuk et al., 2023), and identification of key pheromones such as ascarosides (Choe et al., 2012; Whitman et al., 2021), and small peptides (Chen et al., 2022) in parasite development. Such small molecules may also be detected by the host immune system to flag the presence of parasites. In this setting, host epithelial tuft cells can act as sentinels of helminth infection by detecting molecules released by invading parasites in the gut (O’Leary et al., 2019; Schneider et al., 2018). As tuft cells express sensory receptors associated with taste perception, and signal through a conserved GPCR-associated pathway, we and others have searched for candidate small molecules that may act as a helminth signature at the initiation of infection.

In this study we took a comparative approach to identify small metabolites common to two intestinal nematode species, *N. brasiliensis* which is a widely used rodent model that stimulates an untrammeled type 2 response, and *H. contortus* that is a parasite of sheep and goats globally. Although these parasite species employ different entry strategies, with larvae penetrating through cutaneous and gastric surfaces respectively, in both cases adult worms occupy gastrointestinal niches with similar mucosal immune challenges.

To chart and compare parasite products, we used HILIC for high-sensitivity analysis of small metabolites. Our data compare with studies using alternative analytical platforms, such as gas chromatography-mass spectrometry (GC–MS) or liquid chromatography-MS (LC–MS). For example, all three approaches identified stearic acid as one of the major fatty acids released by parasitic nematodes including hookworms, *Trichuris muris* and *N. brasiliensis* (Wangchuk et al., 2023). Similar to previous studies (Wangchuk et al., 2019b, 2023) common and species-specific molecules were identified.

From our comparative data, we selected 19 molecular species to test in a mouse model of tuft cell induction, administered by oral gavage as previously conducted with succinate that effectively drives expansion of this target cell population (Drurey et al., 2022; Schneider et al., 2018), through the succinate receptor GPR91. While malate showed a low level of induction, we found 10- and 11-carbon fatty acids to be equal to succinate; a previous report had identified bacterial *N*-undecanoylglycine to activate tuft cells, and to do so via the olfactory Vmn2r26 receptor expressed by these cells (Xiong et al., 2022). Medium chain fatty acids (9–14 carbons) bind GPR84, but as far as is known, this receptor is restricted to myeloid lineages such as macrophages and neutrophils (Wang et al., 2006). Future studies will aim to test the role of these receptors in helminth-induced tuft cell hyperplasia, and in guarding the host against helminth invasion. Notably, succinate and decanoic (capric) acid were also reported in secreted products of other GI nematodes, including hookworms and *T. muris* (Wangchuk et al., 2023).

Our HILIC and analysis technique focused on metabolites from conventional pathways, and hence would not have detected substances unique to nematodes. We conducted a directed search for ascarosides, small pheromone molecules associated with nematode communication (Choe et al., 2012; Whitman et al., 2021) that also show interactions with immune responses (Shinoda et al., 2021); we noted ascr#1 from *N. brasiliensis* but not *H. contortus*, perhaps reflecting the requirement for species specificity within the pheromone compartment. Future work on this class of small molecules is likely to identify further key host interactions. Within the standard metabolites, only malate was found to show a low level of activation. However, malate may be converted to succinate via fumarate, which could account for stimulating a low level of tuft cell activation by this compound.

The changes in amino acid levels, both consumption and production, are likely to have physiological effects on host cells. For example, group 2 innate lymphoid cells (ILC2s) are highly dependent on large neutral amino acids such as phenylalanine (Hodge et al., 2023). Release of this amino acid, most notably by *N. brasiliensis*, could potentially enhance ILC2 responses to infection. The differential effect of hypoxic conditions included some interesting results; it is known from microbial and *C. elegans* nematode metabolism that an anaerobic environment promotes succinate production (Del Borrello et al., 2019).

Our approach of oral delivery does have some limitations, in that we could not evaluate molecules with poor water solubility, and had little control over whether substances reached the small intestine in sufficient concentrations, or were converted or absorbed earlier in their journey. Targeted studies to determine the prevailing concentrations of, for example, medium chain fatty acids in the small intestinal lumen would be informative in this regard. Although we based the concentrations of administered molecules to be below any potential toxic levels, they are nevertheless given at relatively high molar concentrations, and nonspecific perturbations of the epithelium may result; in this setting, damage repair pathways may include IL-13 production, giving rise to tuft cell expansion in the absence of any sentinel receptor activation. More broadly, effects from oral administration of test substances does not necessarily show a direct mode of action, for example if molecules are metabolised or converted in vivo, or if they act on microbial populations in the intestinal tract, resulting in gain or loss of microbiome mediators. Medium chain fatty acids such as decanoic (capric) acid have indeed been reported to have anti-bacterial activity that could impact on tuft cell induction. Future studies in organoid culture systems will minimize a number of these concerns and facilitate demonstration of direct activities.

In conclusion, we have identified a second class of small molecules, the 10/11-carbon fatty acids, that can induce tuft cell differentiation in vivo, although their direct action through a specific receptor has yet to be established. Importantly, these results indicate that multiple ligands may have the capacity to act on intestinal epithelial tissues to promote development of secretory lineages including tuft cells; these findings raise the possibility that redundant mechanisms have evolved to detect the presence of helminths, and that additive or combinatorial signaling may be employed to maximise sensitivity and specificity for helminth infection.

## Supplementary Information

Below is the link to the electronic supplementary material.

Supplementary file1 (PDF 133 KB)— Suppl Fig. 1: Ascarosides released by N. brasiliensis adult worms. 200 90 adults were incubated in 1 mL media; supernatants were recovered and extracted in chloroform:methanol:water for MS analysis. Data for mass 276.1276, corresponding to ascaroside #1 (276.1573). Suppl Fig. 2: Comparative intensity of 15 major metabolites released by N. brasiliensis and H. contortus, in same format as Figure 3 B, shown for EBSS (A) and RPMI1640 (B).

Supplementary Table 1 (XLSX 102 KB)

Supplementary Table 2 (XLSX 148 KB)

Supplementary Table 3 (XLSX 1973 KB)

Supplementary Table 4 (XLSX 319 KB)

## Data Availability

Data is provided within the manuscript or supplementary information files.
