# Supplementary figures and images for: Metabolomic and functional analyses of small molecules secreted by intestinal nematodes in the activation of epithelial tuft cells
Source: Metabolomics. 2025 Apr 21;21(3):55. doi: 10.1007/s11306-025-02248-w (PMC12011944; doi:10.1007/s11306-025-02248-w)

Campillo Poveda Suppl Figure 1

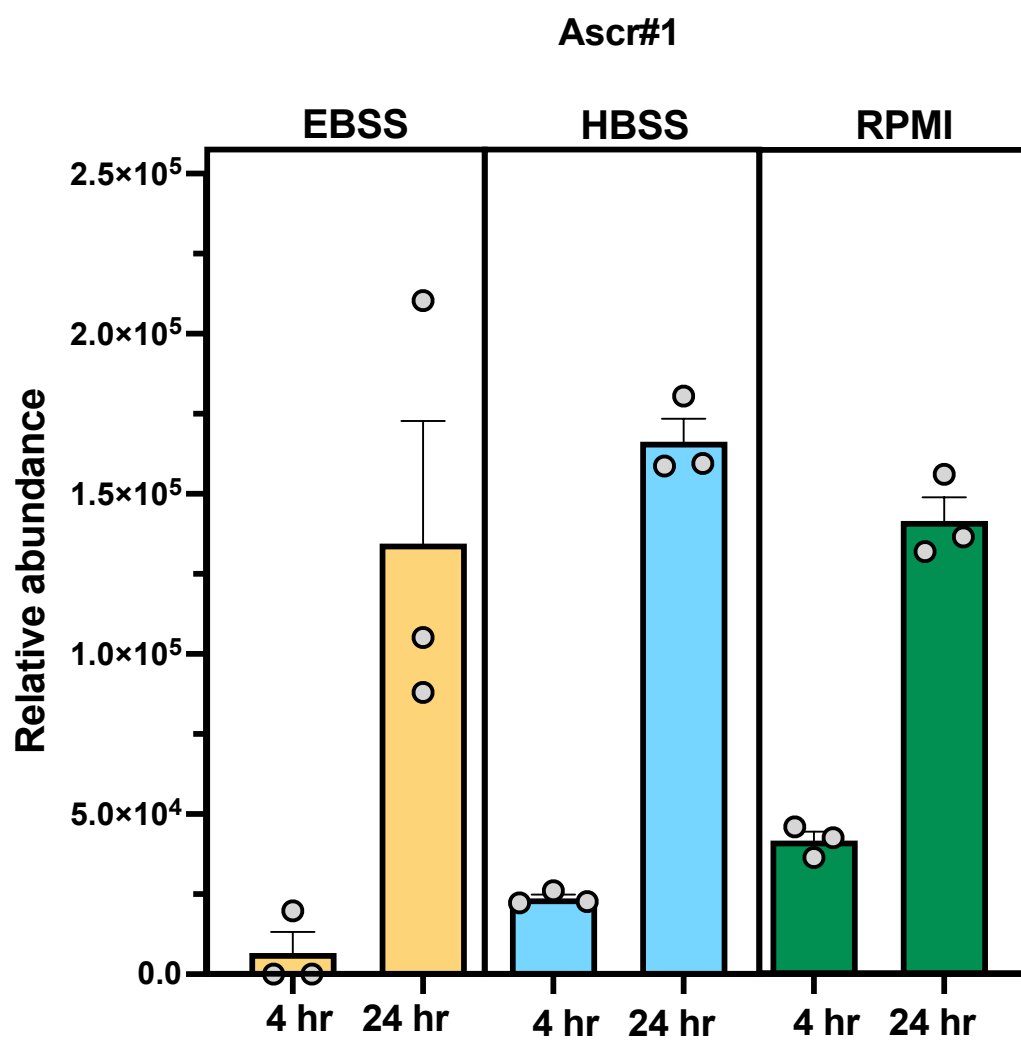

A

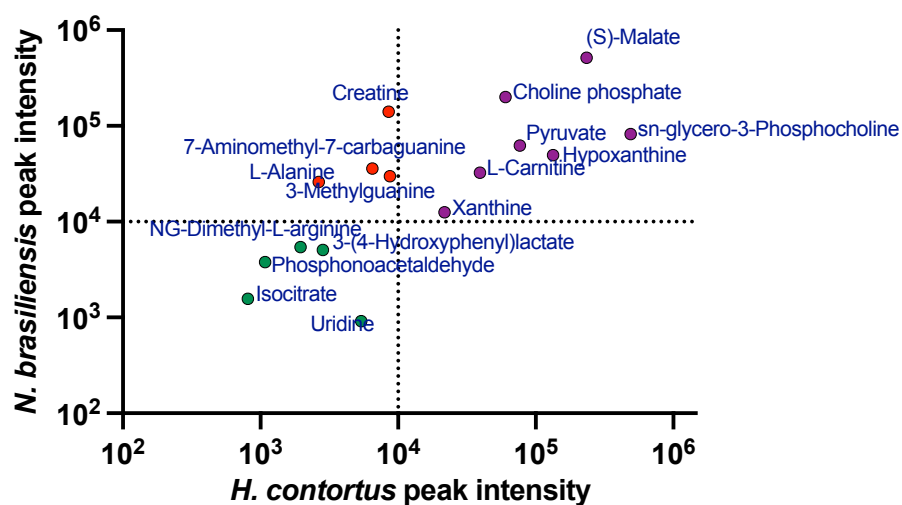

B

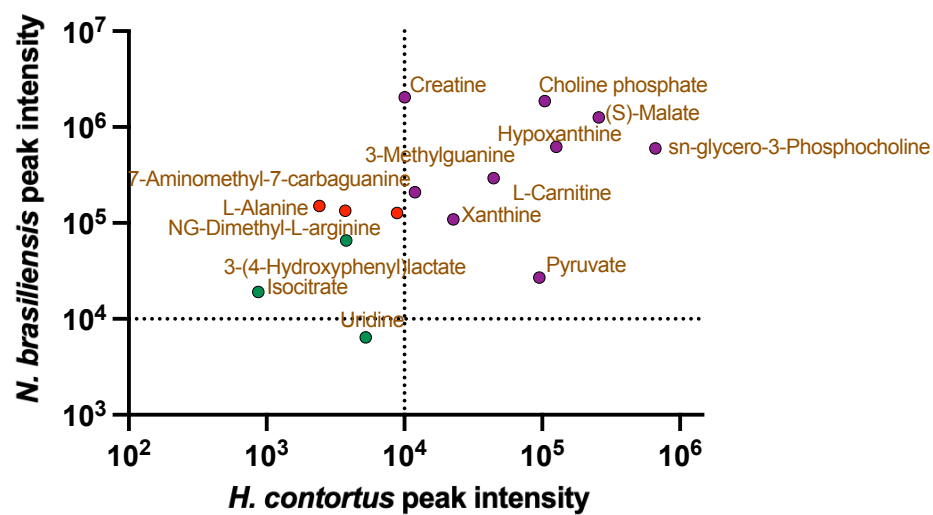

Supplement: Supplementary file 1 — Supplementary file1 (PDF 133 KB)— Suppl Fig. 1: Ascarosides released by N. brasiliensis adult worms. 200 90 adults were incubated in 1 mL media; supernatants were recovered and extracted in chloroform:methanol:water for MS analysis. Data for mass 276.1276, corresponding to ascaroside #1 (276.1573). Suppl Fig. 2: Comparative intensity of 15 major metabolites released by N. brasiliensis and H. contortus, in same format as Figure 3 B, shown for EBSS (A) and RPMI1640 (B). [file 11306_2025_2248_MOESM1_ESM.pdf]
